# Primary Cognitive Categories Are Determined by Their Invariances

**DOI:** 10.3389/fpsyg.2020.584017

**Published:** 2020-12-08

**Authors:** Peter Gärdenfors

**Affiliations:** ^1^Cognitive Science, Department of Philosophy, Lund University, Lund, Sweden; ^2^Faculty of Humanities, Palaeo-Research Institute, University of Johannesburg, Johannesburg, South Africa

**Keywords:** category, invariance, space, object, place, event, number

## Abstract

The world as we perceive it is structured into objects, actions and places that form parts of events. In this article, my aim is to explain why these categories are cognitively primary. From an empiricist and evolutionary standpoint, it is argued that the reduction of the complexity of sensory signals is based on the brain's capacity to identify various types of invariances that are evolutionarily relevant for the activities of the organism. The first aim of the article is to explain why places, object and actions are primary cognitive categories in our constructions of the external world. It is shown that the invariances that determine these categories have their separate characteristics and that they are, by and large, independent of each other. This separation is supported by what is known about the neural mechanisms. The second aim is to show that the category of events can be analyzed as being constituted of the primary categories. The category of numbers is briefly discussed. Some implications for computational models of the categories are also presented.

## What Determines the Categorical Structure of our Perceptions?

The world as we perceive it is structured into *objects, places*, and *actions* that form parts of *events*. We have a strong tendency to be realists, that is, to believe that these categories exist out there in the world. Kant taught us, however, to distinguish between “das Ding an sich” and “das Ding für uns.” According to him, and much of modern cognitive science (e.g., Marr, 1982; Humphrey, 1993; Anderson et al., 1998; Von Glasersfeld, 2005; Hoffman, 2019), we cannot know external reality but only how our minds construct the world. For such a constructivist position, a fundamental question is why our mental constructs end up with categories of objects, places and actions. The answer, as always, should be grounded in the evolutionary mechanisms that have molded our perceptual systems and in how the brain handles the information presented by these systems.

The senses generate an extremely rich and unstructured mass of signals. When trying to understand what happens to the sensory information in our brains, it is standard to distinguish between *sensations* and *perceptions*. Our subjective world is full of colors and patterns that we see, things that we taste and smell, itches, pains, and sensations of cold that we feel. In philosophy such sensations are called *qualia*. The evolutionary value of sensations is that they inform us about what is happening right now to our bodies (Humphrey, 1993).

An individual that also receives signals about what is going on in the world and not only what is happening to its body will be better prepared to foresee the future and thus to survive in a challenging environment. This is the purpose of perceptions. In order to make sense of the sensations, the perceptions result from processes in the brain that reduce their complexity by structuring them into kinds of entities. In this article, I argue that this complexity reduction is based the brain's capacity to identify various types of *invariances* in the sensory signals—invariances that are evolutionarily relevant for the activities of the organism. My aims are, firstly, to explain why places, object and actions are *primary cognitive categories* in our constructions of the external world, and, secondly, how these components generate cognitive representations of *events*.

Traditionally, there are two approaches to the functioning of the mechanisms of our brain that generate the primary categories: (1) nativism: the categories are *innate*; and (2) empiricism: the categories are *learned*. Spelke and Carey (Spelke, 2000, 2004; Spelke and Kinzler, 2007; Carey, 2009) propose objects, actions, space and numbers as “core knowledge domains,” which form the framework of perceptual categories. They defend a nativist position in relation to child development. In contrast, my solution will be empiricist (Gärdenfors, 2018), although I will suggest that the structure of the brain imposes constraints on how the categories are learned. There is thus a nativist element in my analysis, albeit of a different kind than that advocated by Spelke and Carey. Following them, I will also briefly discuss to what extent numbers form another primary cognitive category.

A part of an evolutionarily grounded argument builds on the fact that human (and other mammal's) infants are not born as blank slates (Pinker, 2002). By evolutionary processes, the brain is prepared to pick the most relevant invariances (see e.g., Leibo et al., 2015). As examples of how the brain organizes invariances, the dorsal stream of the cortex handles space representation (the where pathway), the ventral stream generates object representation (what pathway) and the dorsal stream accounts for action representation in (how pathway). Even though these pathways are to some extent neurologically given, the infant must, however, *learn* to identify the invariances that create the most relevant cognitive categories. After the invariances have been learned, the plasticity of the cortex still supports considerable relearning: An amazing example is that a person who is given goggles turning the visual field upside-down, will, after a few weeks, be able to relearn the projection from the visual cortex so that the perceived world is “normal” again (Kohler, 1951).

The strong capacity to detect invariances that the brain has, leads to the crucial question concerning which cognitive categories that are the most fundamental. A central question for the analysis becomes: *Why* are the invariances that determine places, objects and actions cognitively primary?^1^ This is, in a sense, a neo-Kantian epistemological question, seeking the “forms of perception” (“Anschauungsformen”) that generate the framework for more specific categorizations.

By using an analysis in terms of invariances, I will show that each of the categories of places, objects and actions has its separate characteristics and that they are, by and large, independent of each other. A preliminary attempt to identify primary cognitive categories in terms of invariances for space, objects and actions was made in Gärdenfors (2018). That paper dealt with two learning processes: how the primary categories are learned and how concepts that are grounded in the categories are learned. This paper presents a more detailed analysis of the role of invariances and also analyses the categories of numbers and events.

## Extracting Structure: Invariances in Perception

The primary categories build up our perceptual structures. My thesis is that the sensations, at an early stage of the process in the brain, become perceptions that are organized along primary ontological categories, in particular space, objects and actions. By saying that the categories are primary, I mean that they form the fundament from which specific concepts are constructed, for example, places as regions of the space, object categories as determined by specific properties or part-whole relations, etc. Since they are founded in the mechanisms of the human brain, they are also seen as common to all humans.

My approach to perception is in some respects similar to Gibson's (1966, 1979) “ecological approach.” He writes: “The individual does not have to construct an awareness of the world from bare intensities and frequencies of energy; he has to detect the world from invariant properties in the flux of energy” Gibson (1966, 319). A useful metaphor is that the brain *resonates* with the sensory information. (Gibson, 1966, 201) defines an invariant as a “non-change” that persists during change. This definition is not very useful for identifying invariances so I will instead rely on well-known types of invariances, some taken from physics and some from analyses of children's cognitive processes. Following Breidbach and Jost (2006), I outline in this section how a theory of perceptual invariances can explain our primary categories. A central type of perceptual information is what remains invariant when an agent moves through the environment and interacts with objects in it (see also Cutting, 1986).

Unlike Gibson, I take a constructivist position and do not claim that invariances are “out there,” ready to be “picked up” by the brain. In contrast, I view invariances as something that is constructed by various processes in the brain. Not all possible invariances are constructed—only those that are relevant for survival. Over the millennia, evolution has selected the invariances that are most salient for the activities of the organism.

One central notion for the analysis of invariances is *fungibility*^2^, that is, replacements of equivalents. For example, a place remains the same independently of which objects are located at the place. In other words, objects are fungible with respect to places. Similarly, an object remains the same independently of which place it is allocated at, so places are fungible with respect to objects. These two types of fungibility form the main reason why the place and the object categories are independent^3^.

## Space

According to Gibson's approach, the visual field is determined from invariances such as texture gradients, occlusions and visual flow. To a large extent the visual flow is determined by the movements of our bodies. Turning our heads and letting our eyes follow along, for example, leads to vary rapid changes in the image that reaches the retina. However, our brain simultaneously produces a representation of the surrounding space that remains still relative to the direction of our body.

During the first months of life, an infant learns how to coordinate sensory input—vision, hearing, and touch—with motor activities (Thelen and Smith, 1994). The infant engages in “motor babbling” that generates an egocentric representation of space, coordinating it with its actions. As Gibson (1979: 2) writes, “the environment to be perceived […] is not the world of physics but the world at the level of ecology.” The space we perceive can be divided into *peripersonal* space—the region immediately surrounding our bodies (di Pellegrino and Làdavas, 2015)—and *extrapersonal* space, which is the space beyond our reach.

The peripersonal space makes it possible for an individual to see its *field of action*. Moving only the head and not the rest of the body, an individual's potential to act does not change. Since the hand actions of the individual occur in front of the body, it's more efficient if the brain creates a space that is constant in relation to the body direction. The peripersonal representation of space is therefore invariant of the direction of the eyes and the head. The space that is constructed is a three-dimensional space where the body determines its origo and principal direction^4^.

The representation of visual space then expands during the child's development. Firstly, when the auditory input is coordinated with the visual, the represented space extends beyond the child's current visual field to cover the entire surrounding space. The child is then able to direct its attention outside its peripersonal field and it becomes extrapersonal. Importantly, the egocentric representation of space that results from this extension is no longer just visual, but an *amodal* representation based on visual, auditory, tactile, and perhaps even olfactory sensations.

The adult visuo-spatial category should thus be seen as a combination of a peripersonal and an extrapersonal space. The two representations have different basic functions: The peripersonal is used for reaching and interacting with objects, and the extrapersonal for surveillance and navigation (Gallistel, 1990).

There are several experiments supporting that the space category is not an innate structure. It must be learned through *interaction* with the world, where a first step is eye-hand coordination (e.g., Held and Hein, 1963; Agrawal et al., 2015). This process must learn how visual (and auditory) information can be used to create meaningful fields of action. For example, getting a new pair of glasses with stronger lenses changes the conditions for this process. Further experience is required before the brain has construed an adjusted space and can provide the perceptions needed for carrying out precise actions, such as walking down stairs without stumbling.

A second extension of the space representation involves the ability to represent an *allocentric* space. This is an *imagined* space where the location of the individual is no longer a fixed point. The allocentric representation makes it possible for the an individual to abandon the egocentric perspectives and instead imagine how the world looks like from another point of view^5^. The allocentric space representation is not just invariant of eye and head orientation but also of the *orientation and location* of the body. The primary role of the allocentric space is to allow planning for movements through space. Piaget and Inhelder's (1967) three mountain test was developed to determine when children master problem solving using representations of allocentric space. For a survey of how humans represent space, see Tversky (2003).

In the brain, a self-centered representation of location is transformed into an allocentric representation by a network involving the posterior parietal cortex, the medial retrosplenial complex and the hippocampal formation (hippocampus and entorhinal cortex) (Nau et al., 2018). The allocentric representation in the hippocampal formation then projects allocentric coordinates back to guide navigation.

Importantly, by extracting the various forms of invariances, the egocentric and allocentric spaces that are generated considerably *reduce* the complexity of the information that hits the retinas. If the constructed allocentric space were perfectly invariant under rotations and translations (so-called Galilean transformations, Levy-Leblond, 1971), it would follow that the resulting visual space is three-dimensional Euclidean. However, since our movements mainly take place in the two horizontal dimensions, the vertical dimension is less important for our perception. Consequently, our perception of the vertical dimension is “flattened” (Kaufman and Kaufman, 2000).

An important aspect of the representation of space is that it is invariant of *time*. When we move or turn around, we perform rotational and translational transformations of the perceptual input. If these transformations were not invariant over time, it would not possible to use the represented space as a basis for actions. This point was made already by Gibson (1966, 264): “An individual who explores a strange place by locomotion produces transformations of the optic array for the very purpose of isolating what remains invariant during these transformations” (see also Agrawal et al., 2015).

The domain of space can be divided into regions or *places*. The identity of a place is determined by its relation to a set of *landmarks* and not by its location in relation to some fixed coordinate system. For example, from my perspective your location may be in the passenger seat of my car that is moving through the landscape. The landmark is the car that determines the relative places inside it. For an extreme case, consider that the earth is rotating around the sun at a very high speed. Nevertheless, we take the earth to be the landmark and say that Sweden is located in northern Europe.

A place is also, to a large extent, invariant of the objects located there^6^. If somebody else sits in the passenger seat of my car, it will still be the same place. If Sweden, due to severe climate changes, turns into a desert, its identity as a place does not change. As mentioned earlier, we can say that objects are *fungible* with respect to places. Similarly, actions are fungible with respect to places—the identity of a place does not depend on what is done there.

Sometimes, other properties than a set of landmarks are used to identify a place, for example its *function*. For example, in 1988 the Australian parliament moved from its old house to a new one in Canberra. Still one can refer to “the parliament” as a location. A more exotic example is that the entire town of Kiruna in northern Sweden will be moved two miles to the east because there is a risk that the extensive iron mining under the town will lead to a collapse of the ground. New streets will be laid out and many of the historic houses and official buildings will be moved to the new location and, but the spatial relations between the buildings will not be preserved. Still the identity of the town will be preserved for most practical purposes.

## Objects

There are many kinds of objects, but I will focus on physical objects, since they have been the most important in the evolution of our cognitive systems. A central property of physical objects is that they have a *shape* (although it may vary over time). This means that the relative locations of different parts of an object can be described in terms of different types of invariances. For a rigid object, the invariances are total. The directions of the parts may change as the object moves, but all the spatial relations between the parts are invariant. For an object with movable parts such as animals, the relations between the locations within each part is more or less invariant and so are the relative locations of the points where the different parts are connected^7^. For example, the parts of your upper leg don't change their relative distances and the connection point between your leg and your body remains invariant. Johansson (1964) calls this type of invariance the “rigidity principle” that functions as a constraint of the visual process: Whenever equal motions in a series of simultaneous proximal elements are detected, the result is a perception of rigidity. Marr (1982) uses this principle extensively in his representation of shapes (for a computationally implemented model see Zhu and Yuille, 1996).

In addition to rigidity or relative rigidity, there are many other types of invariants that apply to objects. The *size* of an object is, for example, typically invariant—at least over short periods of time. This invariance makes it possible to accurately judge the distance to an object. Murray et al. (2005) show that size invariance has been picked up already in the dorsal retinotopic visual area V3. Another property exhibiting invariance is *color*. For many kinds of objects, for example, different species of birds, the patterns of colors are characteristic features. The absolute colors of objects are not invariant, however, since they vary with the illumination. However, the perceptual *relations* between the colors of an object are, in most cases, invariant (Land, 1977).

Some objects are deformable, for example cushions, towels and doughs. Even though invariances of relative locations are less stable for such objects, the changes of relative locations are still continuous. This is what distinguishes objects from masses. Another general type of invariance is that objects are *cohesive*: if you pull at one end of an object, the other parts will follow. Clouds, flames and shadows are therefore marginal as objects. Leslie (1996) argues that infants just a few months old perceive the world as consisting of cohesive objects that keep much of the same form even when moving.

Clouds, flames and shadows indicate that there are grades of objecthood: they have properties that make them come close to being masses rather than objects. The characteristic distinctions between masses and objects is that masses, such as water and sand, (i) do not have a constant shape, (ii) are variable in size and (iii) are homogenous in material. Linguistically, the distinction shows up in that mass nouns are not countable—one does not say “two sands”—but nouns for objects are^8^.

Neuroscientific support for the thesis about invariances determining the object category is becoming stronger. In particular, Leibo et al. (2015) and Anselmi et al. (2016) present a neural model of object and face recognition based on invariances that builds on the idea that the main task of the ventral stream of visual processing is to compute a “signature for recognition” that is invariant of translations and rotations. They also show that when the relevant transformations have been learned for some objects it generalizes to other objects. For example, if we see a new face in a frontal position, we can accurately predict how it will look like if turned to the side. The grouping of objects is done by their transformation compatibility, that is, the class of transformations that preserve their identity. Another type of support comes from Kriegeskorte et al. (2008) who show that the inferior temporal cortex of monkeys and human share a common code for representing objects, in particular concerning major distinctions such as animate–inanimate and face–body. The response patterns in the cortex form category clusters that match between monkeys and humans.

The perception of objects also involves an extensive reduction of the dimensions of the sensory input. Several computational procedures for dimension reduction have been proposed, for example Principal Component Analysis (Abdi and Williams, 2010) and Multidimensional Scaling (Kruskal and Wish, 1978; Borg and Groenen, 2005). It is not known, however, how similar these procedures are to real brain processes. Wiskott and Sejnowski (2002) have developed an artificial neural network based on “slow feature analysis” that is able to pick up translation, size, rotation, illumination and contrast invariances of objects. From a neuro-cognitive point of view, an interesting feature of the neural network is that the “what” and the “where” components become represented in separate components of the network. This provides indirect support for my hypothesis that the space and object invariances can be separated. The invariances that lead to the dimension reduction, both in Wiskott and Sejnowski's model and in that of Anselmi et al. (2016), show that the dimensional structure that is represented is closely related to a 3D Euclidean space. This is congenial with proposals that the hippocampal formation is not solely used to represent spatial information, but is also exploited to represent other types of conceptual spaces (Eichenbaum and Cohen, 2014; Bellmund et al., 2018).

When describing how infants represent objects, Spelke et al. (1992, 606) suggest the following criteria: (i) *continuity* (objects move in continuous paths), (ii) *solidity* (objects move only on unobstructed paths and therefore different objects do not occupy the same place), (iii) *gravity* (objects fall downwards, if not supported), and (iv) *inertia* (objects do not change their motion abruptly).

Except for solidity, which I have discussed above, these constraints do not concern invariances of objects. The last two are not about objects *per se*, but rather describe the behavior of objects. Furthermore, objects that are *agents* violate the inertia constraint. Surprisingly, the list of criteria proposed by Spelke et al. (1992) does not contain shape, despite the fact that children's categorizations of objects have a clear shape bias (e.g., Landau et al., 1998; Smith and Samuelson, 2006).

A consequence of the representation of the continuity of objects is *object permanence* (Piaget, 1952), which means that objects are represented as being located at the place where they were last perceived, even if they currently do not produce any sensations. This means that the object is represented (imagined) in the inner world as located at a particular place, even if it is not perceived. The ability to keep an object in mind is not innate; human infants acquire it around 5 months of age (which is later than among other animal species) (Baillargeon and DeVos, 1991).

## Actions

The third primary category of our perceptions involves actions. Humans are exceptionally efficient at categorizing actions. For example, it is easy to instantly judge whether somebody is walking or jogging, even if the movements of the body parts are rather similar. Furthermore, only a very limited amount of information is needed to make such a categorization. The efficiency of action perception was shown by Johansson in a series of classical perception studies in the 1950's (Johansson, 1973). The patch-light technique that he invented for analysing biological motion contains no direct shape information. Light bulbs were attached to the joints of actors who were dressed in black and moved in a black room. The actors were performing different actions such as walking, running, and dancing while being filmed. Subjects who then watched the films saw the movements of the light bulbs (but nothing else). They were able to correctly categorize the actions within a few hundred milliseconds.

Experiments of this kind indicate that that seeing the surfaces of the agents performing actions is not necessary for categorizing the actions (Hemeren, 2008). A movie that contains stick figures or only dots moving in the same way is sufficient. These observations give additional support to Johansson's rigidity principle. The question now is what kind of invariances are involved in action categorizations.

Working in the tradition of Gibson, Runesson (1994, pp. 386–387; see also Wolff, 2008) argues that people can directly perceive the forces that generate different types of motion:

“The fact is that we can *see* the weight of an object handled by a person. The fundamental reason we are able to do so is exactly the same as for seeing the size and shape of the person's nose or the color of his shirt in normal illumination, namely that *information* about all these properties is available in the optic array.”

Runesson formulates this as that the kinematics of an action is sufficient to identify the underlying force patterns. For example, the pattern of forces involved in saluting is different from the pattern of forces involved throwing even if the actions are perceptually rather similar. Johansson and Runesson mainly apply their principles to biological motion. I hypothesize, however, that they can be applied to other forms of action as well. I have argued that the brain extracts the invariances that represent the *forces* that generate different kinds of actions (Gärdenfors, 2007, 2014). The process extracting the invariances is automatic: an individual cannot help perceiving the forces (Wolff, 2008; Wolff and Shepard, 2013; Wolff and Thorstad, 2017). Just as for objects, the space of force patterns can therefore be seen as a perceptual category with a unique structure of similarities and defined by its own class of invariances. Of course, the perception of forces is not perfect; people are prone to illusions, just as in all types of perception (Johansson, 1964, 1973).

An example of an empirical study of force patterns it that of Wang et al. (2004). Based on data from the walking patterns of humans collected under different conditions and using the methods of Giese et al. (2008), the force patterns that were extracted were used to calculate the similarity of the different types of walking^9^.

A particular action is, of course, performed by a particular agent (a special kind of object) at a particular place. For the categorization of an action, however, a central invariance is that only the forces, but *not* the individuals or objects performing the action, are involved in the representation of the action. More generally, patterns of forces should be considered since several body parts are typically involved; and several force vectors are consequently interacting. This is analogous to Marr and Vaina's (1982) differential equations for actions. Such force patterns form the invariances that I submit generate the structure of action categories. However, the invariances that apply to actions are neither the same as those for objects, nor for those for space. To wit, the patterns for actions are neither dependent on the location of the acting object, nor on its object properties such as color or weight. This means that the objects and places are fungible with respect to actions and thus that the action category is independent of the object and space categories. In line with the situation for space and objects, the force patterns determined by the invariances involve a considerable reduction in dimensions. However, the empirical data concerning how actions are perceived is still limited so the precise structure of action space should be further investigated.

Human understanding of actions, however, does not only involve physical movements and their underlying forces, but often also the *intention* behind the action. For example, “blink” and “wink” cover the same kinds of physical eye movements, but the second action is intentional. Accounting for the intentionality of actions also involves representations of a *goal space* in the agent that is attributing the intention (Gärdenfors, 2014, pp. 194–197). It might be argued that such a goal space should also be included in among the primitive cognitive categories. The main reason for not counting the goal space to the primary categories is that representing intentional actions presumes the capacity to represent actions. This position is supported by recent experiments by Ganglmayer et al. (2019). In contrast to what has been claimed previously (Woodward, 2009, 2013), their results indicate that 11-12-month-old infants anticipate the movement path rather than the goal of an action.

To sum up: The three basic categories place, object and action are mutually fungible relative to each other. As a consequence these three categories are, to a large extent, cognitively independent: Space can be characterized independently of the objects and actions present; objects can be characterized independently of where they are located and which actions are performed on them; and actions can be characterized independently of where they are performed and who (what) performs them. These mutual invariances support my thesis that they form independent primary categories for our cognitive processes.

Following the strategy in Breidbach and Jost (2006), *sub-categories* can then be identified by adding the relevant invariances that characterize them. I have already mentioned the distinction between rigid and non-rigid objects, where the rigid objects are characterized by all distances between points on an object being invariant over time. Another example is the distinction between agents and non-agents, where agents are characterized as being objects that are capable of exerting forces. This distinction will be relevant for the model of events that will be presented below.

The primary categories show up in the structure of language, in particular in how it divides words into classes. Gärdenfors (2014, 2018) has argued in some detail that semantic representations of nouns build on the category of objects, and that verbs build on actions. Furthermore, many prepositions express spatial relations. Different languages have different word classes, but all of them have means to denote objects, actions, and spatial relations. This universality of linguistic structure is a further indication that these categories are indeed cognitively primary.

## Events

Even though space, object and actions form categorical structures that are determined by separate sets of invariances, it is obvious that there are interactions between these categories. They are all parts of *events*. Therefore, I suggest events as an overarching category for combining different perceptual categories (see also Strickland, 2017). Already Gibson (1979, 100) describes events as primary realities. There is an extensive amount of research on how children's event cognition develops (e.g., Radvansky and Zacks, 2014, Ch. 10; Papafragou, 2015).

**Figure 1:**
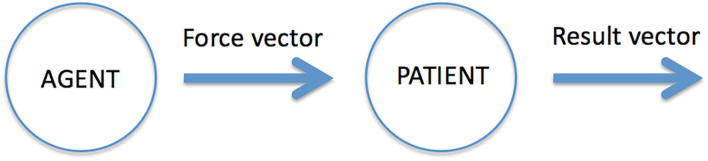
The main components of an event representation.

The cognitive structure of events is relational, gluing together objects, actions and locations. In earlier work (Gärdenfors and Warglien, 2012; Warglien et al., 2012; Gärdenfors, 2014), I have suggested an approach to event categorization based on some geometric notions. The key idea is to represent event structures in terms of conceptual spaces—one for actions and one for results—and *mappings* between these spaces (see Figure 1).

Following the previous section, the action space is represented as a space of forces (or force patterns) acting upon some object. As mentioned above, I view non-intentional actions as primary. Modeling intentional actions would require adding a goal space to represent the aim of the action. The result space of the event represents changes in the properties of the target. This space can therefore be modeled as a vector space where the two ends of a result vector represent the properties of the object acted upon before and after the action^10^. The results of actions are typically changes of location (that is, the space category) or changes of object properties. For example, when Donald pushes the table, the agent Donald exerts a force vector (action) on the table that leads to a change of the position of the table (result). Or in the event of heavy rain undermining a road, the force of the rain (action) leads to a change of the shape property of the road (result). More complicated to represent mathematically are events of breaking or dividing when the object acted upon changes into two or more, and events of construction where different objects are combined into a new one^11^.

A consequence of characterizing an event as a combination of an action space and a result space is that the time domain is not defining for events, but it emerges from the relations between the components of an event. This position contrasts with, for example, Zacks and Tversky (2001) who focus on the temporal structure of events, in particular on how events are segmented. It is often suggested that cognitive representations of events presuppose representing time (Radvansky and Zacks, 2014; Hoerl and McCormack, 2019). For example, Zacks and Tversky (2001, p. 3) write that an archetypical event is “a segment of time at a given location that is conceived by an observer to have a beginning and an end.” If this were correct, time would also be primary category. Of course, the circadian system of our bodies in a sense represents the day and night cycle^12^. However, this system is inflexible and not involved in our representations of events. In fact, there is linguistics evidence that indicates that time is not a primary cognitive category: The abstract time dimension is not used by all human societies but it is the product of cultural systems for measuring time intervals, and hence time is a socio-historical construction (Sinha and Gärdenfors, 2014). For example, the South American language Amondawa does not have an explicit representation of time. This language employs a time interval system that represents seasonal and diurnal events, but it has no calendric terms, including terms such as month and year. Furthermore, children understand events earlier in their development than they understand time as a separate dimension. The model of events presented here does not explicitly represent the time dimension. However, temporality is implicit in the model since actions and events are dynamic entities—they unfold over time.

The action space and the result space represent different categories: forces have a different nature than changes in object properties. In the limiting case when the result vector is the null vector, that is, when nothing changes, the event is a *state*. As can be seen from this two-vector model of events, it combines the three primary categories of objects, actions and physical space into a relational structure: An event can be characterized as a mapping from an action on an object to a result.

In linguistics, the target entity of the event is called the *patient*. The object that creates the action vector is called the *agent*. The concept of an agent thus combines the object category with the action category. There exist, however, events without agents, for example events of falling, drowning, dying, growing and raining. An event may also include other “thematic roles” (Dowty, 1991), such as recipient and instrument, but they are not components of all events.

As for actions, a particular event is, of course caused by a particular agent (a special kind of object) at a particular place. An event category is, in general, invariant of the location where it is performed and on which object (patient) the action is performed. Gärdenfors and Warglien (2012) define an event category as a structure (product space) that represents the mapping from the action space to the result space. An example is the event category of pushing a table, which is constituted by the force (exerted by some agent, human or non-human) on the table resulting in a movement (change in space) of the table.

*Causal relations* can also be represented using the event structure (Wolff, 2007, 2008; Gärdenfors and Warglien, 2012; Gärdenfors, 2020a; Gärdenfors and Lombard, 2020): The action causes the result. Most accounts of causation analyse the relation between the action and the effect as a relation between two events (see e.g., Zacks and Tversky, 2001; Radvansky and Zacks, 2014). In contrast, the model presented here views causation as a relation *within* an event by introducing a distinction between forces and changes of states (cf. Wolff, 2007, 2008, 2012; Wolff and Thorstad, 2017). In contrast to many other theories, causes and effects are not treated as symmetrical entities: they belong to different categories—causes to the forces that are applied on objects and results to change in location (in the case of movements) or in some property of objects (color, size, weight, temperature, etc.).

The characteristic part of an event is the mapping between the force space and the result space. For example, pushing a table sometimes results in the table moving, sometimes not; aiming a dart at the bull's eye sometimes hit it, sometimes not. In such cases the mapping between the force vector and the result vector represents two different events. Gärdenfors et al. (2018) analyse three general constraints on event mappings:

- Larger forces lead to larger results (monotonicity constraint).
- Small changes in the force lead to small changes of the result (continuity constraint).
- Intermediate results are caused by intermediate forces (convexity preserving constraint).

Even though it is not the aim of the article to propose computational models of how various forms of invariances can be used in cognitive systems, the event model lends itself to some recommendations for how such models can be constructed [for more details, see Gärdenfors (2019, 2020b) and Gärdenfors et al. (2019)]. There exist several efficient methods for constructing a computational model of space from video, laser range and other forms of input (see e.g., Wyeth and Milford, 2009). Recent advances in deep learning have also led to good methods for object categorization (see e.g., Zhao et al., 2017). It should be noted that these methods depend on the *appearance* of the objects. For robotic interaction with objects, however, these aspects are not the most important. Gibson (1979, Ch. 8) writes that “what we perceive when we look at objects are their affordances, not their qualities.” In other words, it is what we can *do* with objects that matter, not how they look. Shanahan et al. (2020) discuss this problem. As an example, they take the concept of a “container” that is central to much human interaction with the world. The appearance of containers can vary widely, but it is their affordances that are crucial for how we interact with them. There seems to be no good model of how to capture the affordances of objects from, say, a video stream (Shanahan et al., 2020). As regards actions, they are understudied in robotics. The attempts have focused on the results of actions. For example, the algorithms for learning verb meanings developed by Kalkan et al. (2014) are based on “affordance relations” between entities, behaviors, and effects. Most attempts to computationally categorize actions in terms of manner have been based on stored data, but Gharaee et al. (2017b) present on online, real time algorithm.

There thus exists partly successful work in computer science and robotics that generate models of each of the basic cognitive categories space, objects and action. However, there are very few models of how to combine these models to generate representations of events. The one that comes closest to the approach presented here is Hinaut and Dominey's (2013) model of “reservoir computing.” In Gärdenfors (2020b), I make a programmatic attempt to describe a computational approach to events and illustrate it with a partial implementation, based on reservoir computing, in an iCub robot (Mealier et al., 2016).

Finally, a comment on the relation between event representations and language. Gärdenfors (2014) has argued that a declarative sentence typically describes the main components of an event. This thesis provides an explanation of why sentences are natural units in language. The event structure connects naturally to the core “thematic roles” —agent, patient, recipient, instrument, cause and effect, that help children understand how sentences are constructed and what their meanings are. For example, Papafragou (2015, 338) compares how speakers of Greek and English describe events and she concludes that basic patterns in event perception are independent of the language one speaks. Another example is Fernandes et al. (2006) who show that toddlers already in their third year have an understanding of the abstract categories “agent” and “patient.”

## Number

Another cognitive category is *number*. Even though I do not view it as primary, I will discuss it briefly since it belongs to the core knowledge domains proposed by Spelke (2000, 2004) and Carey (2009). Theories of number cognition distinguish between magnitude (“a large bag of beans”), numerosity (“many sheep”) and number (“five cows”) (Gemel and Quinon, 2019). The underlying cognitive processes are divided into two subsystems that handle approximate magnitudes and discrete numbers respectively (Dehaene, 1996). Non-human animals have an approximate number system that allow them to estimate the relative magnitude of two collections, sometimes with surprising precision (Gallistel, 1990). The discrete number system is used only by humans and it must be learned. There exist human cultures, for example the Amazonian tribe of Pirahã, who do not have a discrete number system (Everett, 2017). Thus, like time, number is a cultural construct and not as fundamental cognitively as the space, object and action categories are. This goes against Spelke's and Carey's position that number is a core knowledge domain.

Nevertheless, approximate as well as discrete numbers are governed by invariances (Harbour, 2014). When judging the invariances that determine the categories of numbers, it should first be noted that number is a property of a *collection*. Collections form an abstract type of objects that can have different properties. Some such properties are shared by physical objects, for example weight and location: “These beans weigh 500 grams.” “The radishes are in the plastic bowl in the fridge.” Many properties are, however, unique to collections: For example, collections can be ordered or unordered, uniform (consisting of the same type of objects) or mixed, dense or spread out. In particular, collections have *cardinality*, that is, they contain a certain number of elements. The cardinality of a finite collection is expressed by a natural number.

Numerical invariances of collections have been studied extensively in developmental psychology (e.g., Gelman and Gallistel, 1986; Fuson, 1988; Sarnecka and Carey, 2008). In a series early experiments concerning “conservation tasks,” Piaget (1952) tested children in order to understand which properties of collections they perceive as being invariant. In one experiment, two equinumerous collections of objects, for example marbles, are placed into two parallel lines that are equally long. Then the objects in one line are spread out. A child that has not understood cardinality will say that there are more objects in the longer line. Failing the Piaget conservation tasks means that a child has not understood that a number is a property of a collection that is invariant of its spatial layout (see Gelman and Gallistel, 1986). In other words, number is fungible with respect to the location of the objects in a collection.

The characteristic invariance of the number category is, however, the *fungibility of objects*: If an object in a collection is exchanged for another object, the collection will still contain the same number of objects. Other properties of collections do not fulfill this criterion: If an object (an apple, say) replaces one of the objects in a uniform collection (of oranges, say), the resulting collection is not uniform any more.

The number of elements of a collection is also, to a large extent, invariant under actions, at least in the sense that independently of what kinds of actions the elements perform (for example, the movements of a football team), their number will still be the same. Similarly, number is typically invariant under actions performed on the objects (as long as the actions do not destroy the objects).

## Conclusion

In this article I have used the notion of invariances to explain why the categories of space, object and action are fundamental cognitive structures. In philosophical terms this is a version of the neo-Kantian program of describing the “Anschauungsformen” of our perception. The analysis of the primary categories in terms of invariances can be seen as an explanation of such forms of perception. As a part of the explanation, an evolutionary perspective connects the categories to the success of the activities of an organism. I have also argued that all three categories are central elements in the more abstract category of events. The analysis of the category of numbers that I have presented indicates that, also for non-primary categories, different forms of invariances can be used to characterize a category.

Although the evidence for the invariances that I have presented in this article comes mainly from experiments with human subjects, the perceptual systems of, at least, mammals are sufficiently similar to warrant the conclusion that space and objects are also primary categories for them. As regards actions (and, consequently, events), the situation is less clear^13^.

The enterprise of identifying cognitively primary categories is not only of philosophical and psychological interest. It leads to new questions to cognitive neuroscience. The most pressing one concerns how the invariances are picked up by the brain (e.g., Nau et al., 2018). Understanding these processes may help understanding the foundations of how we perceive the world. I have presented some results concerning how brain processes utilize invariances in creating cognitive representations, but this field has much more to analyse. New perspectives concerning invariances may be used to generate new hypotheses concerning how the brain handles primary categories and to generate new ideas for the architecture of computational and robotic systems that reason about the world and act in it.

## Author Contributions

The author confirms being the sole contributor of this work and has approved it for publication.

## Conflict of Interest

The author declares that the research was conducted in the absence of any commercial or financial relationships that could be construed as a potential conflict of interest.

## Footnotes

^1^I avoid Spelke's use of “core” knowledge structures [and Carey's (2009) “core” cognition] since it is connected with an nativist position, and instead speak of primary categories (see Gärdenfors, 2018).

^2^This term is borrowed from economics.

^3^Fungibility will also be central in my analysis of the number category.

^4^The space is generally throught to have a Euclidean geometry and be based on a Cartesian coordinate system, but there is some linguistic evidence that *polar* coordinates might give a better description of its perceptual geometry (e.g., Gärdenfors, 2014; Zwarts and Gärdenfors, 2016).

^5^The distinction between egocentric and allocentric corresponds to Gibson's (1966) distinction between “perspective structure” and “invariant structure.”

^6^If a place is determined by landmarks, however, the place will change if the landmarks change (Gallistel, 1990).

^7^When categorizing objects with parts, the relations of the parts can be modeled in a “structure space” (Fiorini et al., 2014).

^8^What counts as a mass noun is to some extent language dependent. For example, “furniture” (mass noun in English) is a count noun in French (meubles) and German (Möbel).

^9^Gharaee et al. (2017a) have applied the force dynamic model in a robotic system that has been constructed for categorizing actions.

^10^Slightly more mathematically, an event can be represented as a product space of these two spaces.

^11^Thom's (1972) work on catastrophy theory presents a general way of characterizing such disruptive changes.

^12^Viera (2020) argues that the circadian system allows us to sense time, but he does not consider the role of the time dimension in cognitive event representation.

^13^One reason for this caveat is that animals have difficulties reasoning about causality that depends on external forces (Tomasello and Call, 1997; Povinelli, 2000; Gärdenfors and Lombard, 2020).
